# Standardised Ileal Amino Acid Digestibility in Field Pea Seeds of Two Cultivars Differing in Flower Colour for Broiler Chickens: Effects of Bird Age and Microbial Protease

**DOI:** 10.3390/ani10112099

**Published:** 2020-11-12

**Authors:** Witold Szczurek, Sylwester Świątkiewicz

**Affiliations:** Department of Animal Nutrition and Feed Science, National Research Institute of Animal Production, 32-083 Balice, Poland; witold.szczurek@izoo.krakow.pl

**Keywords:** field peas, amino acids, ileal digestibility, broiler, age, protease

## Abstract

**Simple Summary:**

The extent to which field peas can replace soybean meal in diets for broiler chickens is limited, and one of the reasons for this may be a lack of careful consideration given to the age-related amino acid availability differences in feed formulations. As the digestibility of amino acids in feeds for poultry is a sensitive gauge of their bioavailability, this study has determined and compared standardised ileal amino acid digestibility estimates for whole (raw) seeds of white- and coloured-flowered pea cultivars fed to young and older chickens (14 or 28 days old) in the presence or absence of exogenous protease in their diet. The results demonstrate that when a digestible amino acid system is used, the coefficients of essential amino acids determined at 14 days of age in low-tannin white-flowered peas are not applicable to the formulation of grower-type feed mixtures containing seeds of coloured-flowered cultivars. The increased digestibility of nutritionally essential amino acids in white-flowered pea fed to bids at both ages and in coloured-flowered pea fed to chickens aged 28 days can be expected from protease supplementation. These results contribute to improved use of peas as an alternative to soybean meal vegetable protein sources in diets for broiler chickens.

**Abstract:**

This study aimed to determine and compare standardised ileal digestibility (SID) coefficients of amino acids (AA) in raw seeds of the white-(WF) and the coloured-flowered (CF) field pea cultivar as sole sources of AA in the diets fed to broiler chickens aged 14 or 28 days. An additional purpose was to check the influence of exogenous protease added to pea-based assay diets on AA SID in birds at both ages. Each assay diet was offered to six replicate pens. On both sampling days, the contents from the lower half of the ileum were collected for determination of the apparent digestibility values. The SID coefficients were calculated using ileal endogenous AA losses determined from birds fed an N-free diet. Results indicated a substantial advantage of WF pea over CF pea as a source of digestible Lys, Met, Cys, His, Ile, Leu, Phe, Val, Asp and Glu for 14-day-old chickens. With the exception of methionine and cysteine, there was no significant difference between these two cultivars in the SID values of AA in 28-day-old birds. The protease increased SID of nutritionally essential AA from WF pea-based diet at both ages, and from CF pea-based diet in chickens aged 28 days. In conclusion, the SID coefficients of indispensable AA determined at 14 days of age in low-tannin WF peas are not applicable to the formulation of grower-type feeds containing seeds of CF cultivars.

## 1. Introduction

Since the soybean meal (SBM) is currently a primary source of protein and indispensable amino acids (AA) in swine and poultry compound feeds, the intensive production of monogastric meats and eggs in the European Union (EU) countries is highly dependent on imported soybean. In order to reduce this dependency and to mitigate the imbalance between the EU domestic supply and demand for plant proteins from the livestock sector, the EU introduces measures to promote and expand home-grown protein crops, especially grain legumes that are adapted to the local agro-climatic conditions [1,2]. According to FAOSTAT data, in the EU, and in Europe as a whole, field pea (*Pisum sativum L.*) is the most widely cultivated grain legume species to produce dry seeds used as both animal feed and human food [3]. The average protein content of Pisum sativum seeds is reported to be 24% of dry matter weight with a wide variation between cultivated varieties, growing locations and harvest year. Pea protein is high in some indispensable AA, such as lysine, arginine and leucine, though low in sulphur-containing amino acids [4,5,6]. Research on the utilisation of peas in diets for young growing monogastrics reveals; however, research on the extent to which peas can replace SBM in these diets is limited [2]. It has been shown that partial substitution of dietary SBM with a high amount of raw peas (200–500 g/kg) may have a negative impact on broiler chicken performance [7,8,9]. This is primarily because of the action of secondary metabolites occurring naturally in the whole seeds (collectively referred to as anti-nutritional factors, ANF) or, as concluded by Nalle et al. [10], due to the lack of careful consideration given to the protein and AA digestibility differences in feed formulations. The principal ANF present in pea protein and responsible for lower digestibility of dietary protein and the impaired absorption of AA includes various proteinase inhibitors and lectins [11]. Condensed tannins (proanthocyanidins) and related polyphenolic compounds present in the hull, as well as α-galactosides and phytates, are the primary non-protein components recognised as ANF in this legume crop [12]. Field peas are often classified according to the colour of the flowers, as *Pisum sativum ssp. hortense* with white flowers (WF) or as *Pisum sativum ssp. arvense* with coloured blossoms (CF). Both of these subspecies are commonly cultivated in Baltic EU countries, including Poland [9,13,14]. The two types differ in their tannin compounds content; WF peas contain small amounts of tannins, while in CF, the contents of these compounds are relatively higher [13,15].

Over the past two decades, intensive breeding programmes have led to an appreciable reduction in the ANF content of legume seeds. However, given that secondary metabolites are exceedingly important compounds of chemical defence against pathogenic microorganisms and herbivores [16], it appears that the implementation of the appropriate nutritional strategies or development of economically justified processing methods that partly remove or inactivate the seed ANF post-harvest should be preferred to genetic manipulation. Several advanced processing techniques, such as extrusion, decortication followed by micronisation and air classification, fermentation or treatment with enzymes and organic acids, have been recently proposed for reducing ANF and thus improving the digestibility of pea protein and the availability of AA [17,18]. However, the extra costs involved in applying these techniques and technologies call into question the financial viability of the use of seeds processed in such ways in poultry feed production [9,19]. Another approach that has been considered as a means of attenuating the adverse effects of some ANF present in legumes is the dietary addition of exogenous enzymes [20]. However, several recent studies examining supplementation of diets containing raw pea seeds (30% or more) with xylanase-based carbohydrase complexes (‘cocktails’) have shown inconsistent effects on the performance and/or nutrient digestibility in broiler chickens, depending on the type of cereal grain (wheat or maize) used in feed formulation [7,21,22,23]. Despite direct and indirect evidence that supplementation with exogenous proteases may improve ingredient quality by reducing variability in proteinaceous feed materials and mitigating negative effects of trypsin inhibitors or lectins [24,25], there are only sparse data available on the effects of mono-component proteases on nutrient digestibility in broilers fed diets containing a considerable amount of peas [26].

It is widely accepted that the digestibility of AA in feeds for poultry and other non-ruminant farm animals is a sensitive gauge of the biological availability of these hydrolysed products from protein. Therefore, if broiler chickens are to be fed with properly balanced diets that ensure an adequate supply of AA for metabolism and growth functions, amassing information regarding their digestibility values from different feed ingredients is of importance. The methodology for determining AA digestibility at the terminal section of the small intestine by sacrificing birds and sampling the ileal contents has gained widespread approval as the most exact approach for assessing the value of dietary proteins for growing broilers [27]. A broad consensus has emerged that apparent ileal digestibility (AID) coefficients should be corrected for age-appropriate basal (diet-independent) endogenous AA losses, i.e., for the ileal flow of AA of non-dietary origin [28], which yields more precise values of the standardised ileal digestibility (SID). A growing body of evidence has indicated that the AID and/or SID of particular amino acids in certain cereals and protein-rich feed materials increase with the advancing age of broiler chickens [29,30,31,32], and this suggests that credible ileal digestibility estimates for practical feed formulation should be determined using birds of an age close to the given feeding phase. To our knowledge, however, no published data are currently available concerning ileal digestibilities of AA in whole pea seeds determined in separate studies involving broilers at various ages or in experiments with chickens younger than three weeks. The most recent, extensive and well-described dataset (published in accordance with standard academic practices) that presently exists regarding ileal AA digestibility in feedstuffs important in broiler nutrition [33] provides SID values calculated from the literature data on AID in birds aged 21–42 days and combined for different varietal types of field peas.

Hence, the main purpose of this study was to compare standardised ileal AA digestibility estimates for raw seeds of white- and coloured-flowered field pea cultivars fed to broiler chickens aged either 14 or 28 days. The basis for choosing these ages was from previously established differences in the apparent digestibility of AA in several common feed ingredients [29]. Another, practical, reason behind this choice was that, in multi-phase feeding programs recommended by breeding companies, higher proportions of raw materials potentially burdened with a high amount of ANF are usually included in grower, and finisher diets, offered from 11 or 25 days of age, respectively. The response of a semi-purified pea-based diet to the addition of mono-component serine protease included at recommended concentration was also examined.

## 2. Materials and Methods

The present experiment was carried out at the experimental poultry farm of the National Research Institute of Animal Production (Balice, Poland) and complied with the EU guidelines for the treatment of animals, including the protection of animals used for scientific purposes [34] and the rules for the protection of farmed animals at the time of slaughter [35]. Since no invasive procedures (i.e., causing pain, suffering or lasting harm) have been planned/performed on the broilers, and all were killed solely for the purpose of using their intestines, according to the Polish law (Dz. U. 2015 poz. 266) no explicit approval from an ethics committee was needed before undertaking the research.

### 2.1. Test Pea Seeds and Experimental Diets

Commercial grade certified spring field pea seeds of the white-flowered (*ssp. hortense*) Tarchalska and coloured-flowered (*ssp. arvense*) Milwa cultivars were selected for the experiment performed in 2018. Both pea cultivars were grown at the Polish Plant Breeding Stations located in the middle west region of the country and harvested in 2017. Before inclusion into the diets, the whole seeds were stored at 4 °C and then ground using a cutting mill (Pulverisette-15; Fritsch GmbH, Idar-Oberstein, Germany) fitted with a 2 mm sieve. In conformity with the basic principle of determining AA digestibility by the ‘direct’ method, the peas served as the sole source of AA in the semi-purified assay diets formulated to contain similar amounts of crude protein (CP; N × 6.25). Additionally, a nitrogen-free diet (NFD) was formulated to determine basal ileal endogenous amino acid (IEAA) flows. The IEAA flow values from birds fed the NFD were used to standardise the apparent digestibility coefficients. All experimental diets were offered as dry mash. The assay diets consisted of 81 and 71% of the seeds Tarchalska and Milwa cultivars, respectively, to achieve CP level of approximately 16%, with the remainder of the diet composed of maize starch (Sigma-Aldrich, S 4126) and dextrose (Merisweet 200; Brenntag Poland Ltd., Kędzierzyn-Koźle, Poland) at a ratio 1:1. The NFD was based on dextrose and maize starch and had 5% purified cellulose (Sigma-Aldrich, C 8002) serving as a fibre source, while NaHCO_3_, KCl, K_2_CO_3_ and NaCl were included to adjust the dietary electrolyte balance to the value of about 108 milliequivalents (mEq) per kg diet [28]. All the diets were balanced in terms of calcium and phosphorus and identically fortified with vitamins and trace minerals by using a commercial premix. Rapeseed oil was added to reduce dustiness, and chromium oxide (CAS 1308-38-9) was included as an indigestible marker at 0.5% of the diet. The ingredient composition of the diets is shown in Table 1.

The assay (pea-based) diets were fed either unsupplemented or supplemented with microbial serine protease included on top of the recipe, resulting in two dietary treatments within a given pea variant. The serine protease used in the experiment is an active agent of a solid commercial preparation (Ronozyme^®^ ProAct CT) produced by submerged fermentation of a genetically engineered strain of *Bacillus licheniformis* (DSM 19670). The enzymatic activity for this protease is measured in PROT units, with 1 unit defined as the amount of enzyme that liberates 1 μmol para-nitroaniline (pNA) from 1mM Suc-Ala-Ala-Pro-Phe-pNA substrate per minute at pH 9.0 and temperature 37 °C [36]. The commercial product was obtained from DSM Nutritional Products Ltd. (Mszczonów, Poland). Based on the guaranteed analysis (75,000 PROT units/g) of this product, the protease was included at the recommended concentration of 15,000 PROT units/kg feed provided when the product was added at 200 mg/kg diet.

### 2.2. Birds and Housing

One-day-old, feather-sexed Ross 308 chickens (600 birds, sex ratio 1:1) were distributed into slatted-floor metal cages (length × width × height = 120 × 65 × 50 cm) arranged in a four-tier battery in an environmentally controlled broiler house. Each cage was equipped with four nipple-cup drinkers and a trough feeder along the front of the cage. Manure collecting belts were located between each tier of cages. The male and female chicks were kept separately (20 birds/cage from 1 to 7 days and eight birds/cage from 8 to 21 d) and fed a commercial maize-soybean meal diet in crumble form (220 g/kg CP) until they were assigned to the experimental diets. Room temperature was maintained at 32 °C during the first four days and gradually reduced to 21 °C by the end of the experiment (day 28). The lighting cycle was set to provide continuous fluorescent lighting until 5 days old and then switched to 18 h light and 6 h dark per day. The birds had free access to feed and water throughout the feeding period and were continuously observed for health status. At 8 days of age, 360 chickens of similar body weight (average weight across both sexes 243 ± 22 g) were selected and allotted to 30 clean cages (six males and six females per cage) for the collection of ileal digesta at 14 days of age. At 22 days of age, the remaining 240 birds (average weight 1005 ± 31 g) were distributed to 30 cages each containing four males and four females for the collection of digesta at 28 days post-hatch. At each collection time, a single cage was treated as a replicate. Based on a completely randomised design, the cages were allotted to five experimental diets (one NFD, two pea-based diets containing no enzyme, and two pea-based diets supplemented with protease), with six replicate cages per diet.

### 2.3. Digesta Sampling and Processing

The procedures used for ileal digesta collection were adapted from those recommended by Ravindran et al. [37]. The birds from both age groups consumed the experimental diets (available *ad libitum*) for five consecutive days. At sampling (14 and 28 days), the chickens had free access to diets for at least 20 min before slaughter to ensure complete filling of the small intestine. All the chickens were stunned cage-wise using a head-only electrical method with a current of 250 mA and 400 Hz (STZ6 apparatus, Koma Ltd., Wilkanowo, Poland) and exsanguinated by a qualified person. Directly after the slaughtering process, the abdominal cavity was opened, and the ileum (portion of the small intestine extending from the vitelline diverticulum to approximately 4 cm proximal to the ileo-caecal junction) was removed. The contents of the lower half of the ileum were gently flushed with deionised water, pooled within a cage (12 or eight birds) and deep-frozen (−20 °C).

### 2.4. Sample Preparation and Chemical Analyses

Prior to chemical analyses, the pooled samples of ileal digesta were freeze-dried and ground in a mortar and pestle to a fine powder. Raw pea seeds and pea-based assay diets were ground using a variable-speed rotor centrifugal mill (Pulverisette-14; Fritsch GmbH, Idar-Oberstein, Germany) to pass through a 0.50 mm diameter sieve for determination of dry matter (DM) and crude nutrients (protein and fibre), and a sieve of 0.08 mm diameter for AA analysis. The proximate and amino acid analyses of the samples were performed according to AOAC methods [38]. Dry matter content was determined on the test seeds, experimental diets and lyophilised digesta by drying the samples at 103 °C for 5 h in a forced-air oven (method 935.29). The pea samples were analysed for total nitrogen (N) by the Kjeldahl procedures (method 955.04D) and crude fibre using the conditions described for method 978.10. The AA content of the seed, diet and ileal digesta samples was determined based on the method 982.30E (steps a and b). Before the analyses, all the samples were hydrolysed under nitrogen with 6 N HCl at 110 °C for 24 h. For cysteine and methionine determination, the acid hydrolysis was preceded by overnight cold performic acid oxidation and neutralisation with hydrobromic acid. Tryptophan was not analysed. Amino acid concentrations in the hydrolysate were determined by HPLC after post-column derivatisation with ninhydrin reagent using an AAA 400 analyser (Ingos Ltd., Prague, Czech Republic) and ChromuLan ver. 0.8.1 software (PiKRON Ltd., Prague, Czech Republic) for peak integrations. The chromium contents in the diet and ileal digesta samples were measured in triplicates by the method of Saha and Gilbreath [39] with an Avanta Sigma (GBC Scientific Equipment Pty Ltd., Braeside, Australia) atomic absorption spectrophotometer after wet mineralisation of the samples in HNO_3_/HClO_4_ (1:1.5) mixture. The seed samples were analysed for trypsin inhibitor activity (TIA), total phenols (TP) and condensed tannin (CT) contents. TIA was determined spectrophotometrically at a wavelength of 410 nm with the use of benzoyl-L-arginine-p-nitroanilide (L-BAPA) as the substrate [40]. The TP contents were analysed as described by Singleton et al. [41] using the Folin-Ciocalteu colourimetric method, and the results were expressed in terms of tannic acid equivalents on a dry weight basis. CT was determined by the vanillin-sulphuric acid assay according to Kuhla and Ebmeier [42], and the results expressed as g of catechin equivalents.

### 2.5. Calculations and Statistical Analysis

The coefficients of apparent ileal digestibility (AID) of amino acids were calculated from the formula, whereby the chromium and amino acid concentrations are expressed on DM basis:

AID = 1 − ((Cr in diet × AA in digesta)/(Cr in digesta × AA in diet))

The IEAA flows (losses) were determined in broilers fed the NFD using the following formula:

IEAA (mg/kg of dry matter intake, DMI) = (Cr_d_/Cr_i_) × (AA_i_)

where Cr_d_ is the concentration of chromium in the diet (g/kg DM); Cr_i_ is the concentration of chromium in the ileal digesta (g/kg DM output); AA_i_ is the concentration of amino acid in the ileal digesta (mg/kg DM output).

Apparent digestibilities [Tables S1 and S2, Supplementary Material] were transformed to the standardised coefficients (SID) by correcting AID values for ileal endogenous AA losses for the respective age groups of chickens.The SID was calculated using the following formula:

SID = AID + (IEAA, mg/kg DMI/AA in diet, mg/kg DM)

Statistical evaluation of the results was conducted in accordance with the GLM procedure of Statistica^®^ ver. 12 software package (StatSoft Inc., Tulsa, OK, USA). Pooled digesta samples (one per dietary replicate) served as the experimental unit. Normality of distribution was checked using the Shapiro-Wilk test, and the Levene’s test indicated that the variances for all comparisons were equal. Using an alpha level of 0.05 as a criterion for statistical significance, all SID data were analysed by three-way ANOVA with a 2 × 2 × 2 factorial arrangement of treatments. Age of broilers (A): 14 or 28 d, protease in the diet (P): absence or presence, and pea variant (V): white-flowered (WF) or coloured-flowered (CF) were tested for main effects and all interactions, and a simple contrasts were additionally used to compare the interesting differences between pea variants in chickens of both age groups fed protease unsupplemented diets. Because the significant main effects and V × A, and V × P interactions were noted, the subsets of data for WF and CF cultivars were analysed individually. A single degree of freedom contrast (SDFC) was applied to elucidate the overall effect of independent variables (chicken age and protease supplementation), and their level combinations (treatment) means within each of the two pea variants. All contrasts were considered to be significant at *p* < 0.050. To assess the effect of age on the ileal EAA losses, the respective data for days 14 and 28 were compared using a two-sample *t*-test.

## 3. Results

### 3.1. Chemical Composition of Pea Samples

The amounts of analysed crude nutrients, amino acids and anti-nutritional substances in the test peas are presented in Table 2. The seeds of the WF Tarchalska cv. had somewhat lower contents of crude protein and total AA compared to the CF Milwa cv., the latter mainly due to the lower concentration of glutamic acid and aspartic acid. In comparison with WF, the CF pea seeds contained more crude fibre and had over fourfold higher concentrations of phenolic compounds (expressed in tannic acid equivalents) and condensed tannins (expressed in catechin equivalents) in their dry matter. The examined pea variants did not differ in the value of trypsin inhibitors activity, expressed as the amount (mg) of pure trypsin inhibited per g DM of the material.

### 3.2. Endogenous Amino Acid Losses

As shown in Table 3, except for Cys, Ile, Met, Pro and Thr, the ileal endogenous amino acid and total AA flow was greater (*p* < 0.001 to *p* = 0.025) at day 14 compared to the values determined at the age of 28 d. The total amino acid flow on day 28 was 81% of the respective flow on day 14. The amino acids with the greatest flow were Glu, Asp, Thr and Leu, whereas Met, His and Cys were the least abundant ones in the EAA pool at both 14 and 28 days of sampling.

### 3.3. Standardised Ileal Digestibility of Amino Acids

Standardised ileal AA digestibility data for the test seeds, as affected by pea variant (cultivar), broiler age, and dietary protease, are presented in Table 4. Results from the three-way ANOVA showed that, regardless of age and protease supplementation, the effect of pea cultivar was statistically significant for Glu, Leu, Met and Cys (*p* ≤ 0.005), and for the average digestibilities of 17AA and IAA, with differences in the SID values in favour of the WF Tarchalska cv. The main effect of protease on the SID was found to be significant for all AA evaluated (*p* = 0.000 to 0.048), with birds fed protease supplemented diets having an increased SID coefficient. The effect of age was significant for seven individual AA (Asp, Glu, Gly, Ile, Lys, Pro, Thr) and for the mean digestibility estimates of all AA (17AA) and nine indispensable AA (IAA), where 28-day-old broilers had higher SID (*p* ≤ 0.030) compared with their 14-day-old counterparts. Despite the significant overall (main) effects of the explanatory variables, no significant three-way interaction was observed for any of the AA. However, the significant interactions were found between pea variant and age of broilers for Arg, Asp, Glu, Ile, Leu, Lys, Phe and Pro, and between pea variant and protease for Asp and Ile.

When values determined without supplementary protease were compared using simple contrasts (Table 5), there were marked (*p* < 0.0001 to 0.048) differences in favour of the WF Tarchalska cv. in the SID coefficients for ten individual AA in younger chickens, including seven indispensable (His, Ile, Leu, Lys, Met, Phe and Val) and three dispensable AA (Asp, Cys and Glu). At 28 days of age, the SID of sulphur-containing AA was also higher (*p <* 0.01) in the seeds of WF compared with CF pea. Further detailed analysis of the AA digestibility data for the examined peas using SDFC procedure indicated that in the case of the WF Tarchalska cv. (Table 6) there were no significant differences in the SID coefficients of nine AA (Ala, Asp, His, Ile, Met, Phe, Ser, Tyr, Val). Regardless of age, dietary protease improved the digestibility of Arg, Lys and Thr, as well as the SID values, averaged for all 17 AA and for 9 IAA. The significant positive effect of an enzyme was also observed in 14-day-old chickens for Leu and Pro, and in 28-day-old broilers for Cys and Gly (contrasts *p <* 0.050). In this approach, to compare the treatment combination means within the CF Milwa cv. (Table 7), the SID of all AA (except for Cys) was higher (*p <* 0.050) for 28-day-old birds fed protease-supplemented diet than for 14-day-old chickens fed the assay diet without enzyme. Protease significantly improved (contrasts *p <* 0.050) the SID of His, Thr, Tyr and Val in 28-day-old birds. But there were only numerical differences, resulting from its addition, in the SID of the individual AA in 14-day-old chicks, as well as between younger birds (regardless of dietary protease) and older broilers receiving the unsupplemented assay diet (contrasts *p* > 0.050).

## 4. Discussion

Interactions between environmental factors associated with year (climate) and cultivation area (site) are known to have unforeseeable effects on the nutrient composition and the level of ANF in field peas [4,5,43]. Against this background, it is not surprising that the CP and crude fibre amounts in seeds of the coloured-flowered Milwa cv. tested in the current experiment were higher compared to the values of 236 and 59 g/kg DM, respectively, reported for the same cultivar from 2011 harvest year [13], and that in seeds of the Tarchalska cv. the contents of these components were lower than those shown by Zaworska et al. [44] for pea of the same variety harvested in 2014. On the other hand, the concentration of condensed tannins in the CF Milwa cv. was much higher, whereas in the WF Tarchalska cv., it was almost twofold lower than those recorded by the above-mentioned authors in seeds produced by these cultivars in 2011 and 2014, respectively. However, the lower levels of CT (catechin equivalent) and TP (tannic acid equivalent), as well as crude fibre content of Tarchalska pea in the present study, were in good agreement with the results of many other authors who reported that these substances are present in considerably lower amounts in whole seeds of WF pea cultivars than in the CF ones [5,6,13,15,45,46]. This, in turn, can be explained in large part by a greater proportion of seed coat, rich in polyphenolics and fibre, to total seed weight in the CF cultivars compared to the WF cultivars [15,45]. It is worth noting that the lack of difference in trypsin inhibitor activity between peas tested herein confirms observations from previous studies on the chemical composition and nutritional value of Polish and Latvian pea cultivars with diverse flower pigmentation [13,15,45]. Those studies have also demonstrated that regardless of flower colour variant mean TIA values, expressed in terms of the mass of pure trypsin inhibited per g DM of seed sample, may range widely from 0.45 mg [13] to 3.40 mg [15].

The IEAA flows determined with the NFD feeding method in broiler chickens aged from 21 to 42 days have been reported by many authors, but there is considerable variation within the same age across individual studies [28,47]. On the other hand, there is evidence showing that the basal endogenous AA losses (NFD method) in broiler chickens are age-dependent. An approximately two-fold higher IEAA flow in birds aged 5 days, relative to those at day 15 or day 21, was found by Adedokun et al. [48], and the authors concluded that the observed difference could have resulted, inter alia, from the increased rate of digestion and absorption of mucin glycoproteins—a major source of AA present in endogenous flow—with advancing age. In a more recent study with Ross 308 males, flows determined by NFD for all AA except for Cys were also found to be significantly greater in 10-day-old chickens compared to the values obtained with birds at 24 days of age (325 vs. 161 mg/kg DMI, respectively, for Lys as an example) [31]. This suggests that using basal flows of AA of endogenous origin determined with birds of a particular age to correct the AID estimates for broilers of different ages may reduce the accuracy of the SID coefficients. From among many factors affecting IEAA losses in chickens that are potentially responsible for the discrepancy in values between and within laboratories, the level of dietary electrolyte balance (DEB; calculated as the mEq of Na^+^ + mEq of K^+^ − mEq of Cl^−^ from electrolyte salts, including choline Cl, added to the NFD) may play an important role [28]. Using N-free diets formulated with two levels of DEB (108 and 219 mEq/kg) Adedokun and Applegate [49] found that Arg, Ile, Leu, Phe, Val, Ala, Glu, Gly, Pro and Tyr secretion into the gut increased (*p* ≤ 0.05) with an increasing level of DEB and that mean IEAA loss of the nine indispensable AA in broilers fed the low-DEB diet was 20% lower than that in birds fed the high-DEB diet. Given the foregoing, it is very probable that the difference in DEB was the main reason for notably higher endogenous AA flows of 14-day-old chickens in the current study (raised on an NFD with finally calculated mEq value of 106) compared to the flows previously determined in our laboratory (3663 mg/kg DMI for the total IEAA loss) from birds at the same age fed an NFD with calculated mEq value of eight [50]. It seems that in the case of older birds (28 d), the level of DEB (106 mEq/kg NFD) could also have been a factor explaining the inconsistency between IEAA flows determined in this experiment and the data reported by others. For instance, from a study involving 28-day-old male broilers fed an NFD composed of maize starch and dextrose as major energy sources and with calculated mEq value of 218 per kg of diet, Adebiyi and Olukosi [51] reported the total IEAA flow to be 5414 mg/kg DMI, which was higher by as much as 46% compared to the corresponding quantity in the current investigation.

The current results obtained from feeding protease-unsupplemented assay diets showed a clear advantage of the WF Tarchalska cv. (with CT content of 0.14 g/kg DM) over the CF Milwa cv. (with CT content of 0.63 g/kg DM) as a source of digestible (SID) lysine, methionine, cysteine, histidine, isoleucine, leucine, phenylalanine, valine, aspartic acid and glutamic acid for 14-day-old broiler chickens. While the digestibility values of most AA did not differ significantly, in terms of the standardised ileal digestible amino acids concentrations, as an indication of the quantities of AA that are potentially available to the broilers aged four weeks (SID coefficient of AA for WF or CF at 28 d × AA content in WF or CF, g/kg of DM), the relative similarity of the tested pea cultivars is not apparent. Until recently, very few experimental trials with broilers have been devoted to the determination of the SID estimates of AA in peas as the only source of dietary amino acids (‘direct’ method), all of which were performed on chickens aged between 21–30 days, and the investigated pea variants were white-flowered cultivars [52,53,54]. Furthermore, with the exception of one paper reporting the apparent digestibility coefficients determined in three-week-old broilers by the ‘substitution’ method [13], there appear to be no other peer-reviewed reports that compare ileal digestibility of AA in peas differing in flower colour. Noteworthy is the fact that, with only slight differences, the SID estimates of AA at 28 days reported herein for Tarchalska seeds are similar to those determined for the Polish WF pea (cv. Piast) in the previous bioassay performed in our laboratory with birds aged 30 days [52]. In two independent trials evaluating the UK-grown WF peas with low TIA levels (Mascara and Prophet) for the digestibility of AA in 28-day-old broilers, Masey O’Neill et al. [54] obtained average SIDs of indispensable AA that were quite close to those determined in the present study for the Tarchalska cv. with birds at 14 d, e.g., 0.880 (Arg), 0.860 (His), 0.870 (Lys), 0.79 (Thr). In that study, however, the apparent AA digestibilities were standardised with the basal IEAA loss values compiled from the data that had previously been determined by others in chickens aged 35–37 days. Using 21-day-old male broilers, Bandegan et al. [53] reported surprisingly high SID coefficients of indispensable AA (average value of 0.910; from 0.888 for Val to 0.942 for Arg) in five samples of unspecified pea cultivars derived from different locations in western Canada. These values are much higher than values found for both cultivars and sampling days in the present study and those reported by the aforementioned British research team [54], but unfortunately, the authors did not provide any analytical results regarding the specific ANF contents of test peas.

The reduction in protein or/and AA digestibility in diets containing tannins is attributed to the binding of dietary tannins and feed proteins, and the complexation of tannins with endogenous enzymes [12,55]. In poultry, namely adult roosters and hens and finishing broilers, reports of this adverse effect of tannins from field peas are mostly based on assays performed using whole seeds with relatively high concentrations of tannins (3.6–12.1 g/kg DM) predominantly measured by the amount of tannic acid [6,45,56]. Yet, there is evidence that even very small amounts of condensed tannins within seeds (<1 g/kg DM) can reduce ileal digestibility of their AA in young broiler chickens. Recently, Hejdysz et al. [13] conducted a study to evaluate the nutritive value of three CF (including Milwa) and two WF (Cysterski and Muza) modern Polish pea cultivars, involving the determination of the AID of indispensable AA (except Met) in 21-day-old birds fed a basal diet substituted by 20% (*w*/*w*) of the examined pea. The digestibility of all analysed AA in the two WF cultivars was shown to be significantly higher (by at least 8% points) than in Milwa pea, which, in the case of Cysterski cv., was obviously related to a threefold lower concentration of condensed tannins (0.05 vs. 0.16 g catechin equivalents/kg DM). This observation fits quite well with the current results for 14-day-old chickens. However, it is surprising that for both flower colour pea variants (despite their negligible level of TIA) the authors reported extremely low average AID coefficients of lysine (0.530 for CF and 0.606 for WF), threonine (0.615 for CF and 0.709 for WF) and histidine (0.439 for CF and 0.507 for WF). These values differ considerably not only from the AID values determined in this study (Tables S1 and S2, Supplementary information file) but were also distinctly lower (even up to 40% points) than those determined by a similar ‘substitution’ method for four Australian pea cultivars [57]. It remains rather unclear why, in the present study, substantially higher CT and TP concentrations in Milwa seeds did not contribute to a significant drop in the SID of the majority of AA (compared to the Tarchalska cv.) in broilers aged 28 days. But one of the possibilities for this observation might be due to the fact that older chickens are less susceptible to disturbances in intestinal wall morphology (e.g., destabilisation of enterocytes) in response to dietary tannins at moderate levels (<1 g per kg diet) [58]. However, it should be stressed, that the SID coefficients of sulphur-containing AA determined in chickens at this age for Milwa pea seeds were much lower (*p <* 0.001) than those for Tarchalska. Longstaff and McNab [59] reported similar observations in 25-day-old broilers fed maize-SBM based diets containing faba bean hulls high in condensed tannins, and a large reduction in the digestibility of methionine and cysteine attributed to the increased excretion of inactivated enzymes. Research by Ahmed et al. [60] with four-week-old chickens demonstrated that condensed tannins induce pancreatic hypertrophy and higher trypsin and α-amylase activities in pancreatic tissue, but precipitate these digestive enzymes in the gut lumen, most likely implying enhanced endogenous AA losses. Since pancreatic enzymes are rich in Met and Cys [55], the greater presence of these AA in the intestinal contents can directly result in their lower digestibility values. Mansoori and Acamovic [61], who used broiler males to examine the effect of increasing level of tannic acid (TA) as a model tannin on the endogenous losses of individual AA, found the excretion of methionine and cysteine to be 2.1- and 1.4-fold higher, respectively, in glucose-fed birds dosed orally with 3 g of TA.

The results of the present investigation showed that across the two pea cultivars tested and both ages of birds, all the indispensable and dispensable AA had elevated SID values when the assay diets were supplemented with protease. In the case of cv. Tarchalska, the serine protease used in the current study led to significant improvements in the digestibility of Arg, Lys and Thr, and in the average SID of all IAA in both age groups of broilers. The lack of statistically confirmed response of 14-day-old birds to this enzyme at the applied dose regarding the SID of a specific AA in the CF Milwa pea, as opposed to the Tarchalska cv., is likely due to the resultant effect of higher concentration of protein-precipitating tannins and probable reduction of the activity of pancreatic proteases in the presence of their exogenous counterpart in the intestine of young chickens [62]. It could be speculated that the higher α-galactoside content of Milwa seeds compared with those of cv. Tarchalska might have also contributed to the lower efficacy of the enzyme product used in chickens at a younger age. It is suggested that the presence of α-galactosides causes an increase in the osmolarity in the small intestine and affects the flow behaviour of nutrients and enzymes in the intestinal lumen [63]. Unfortunately, the current study did not determine the concentration of these oligosaccharides in the test peas.

Only a few extensive studies with broilers have been conducted that were aimed at determining the ileal (apparent) amino acid digestibilities of diets composed of plant-based ingredients in the presence or absence of the same mono-component protease as that used in this experiment [64,65,66]. These studies concluded that responses to this specific protease supplementation could be divergently affected by the choice of feed materials used, their proportions and the enzyme inclusion dose. When supplemented at the recommended level (15,000 PROT units/kg), this protease had no effect on the amino acid AID determined with 35-day-old-broilers for a diet based on wheat (32%), maize (30%), SBM (26%) and rapeseed meal (4%) [65], did not influence or decreased AA digestibility in chickens aged 21 days fed maize (56%)-SBM (37%) based diet [66], but increased significantly the digestibility of Arg, Lys, Thr, Val, Asp, Cys and Ser in 22-day-old birds receiving the low protein diet based on maize (65%) and SBM (23%) [64]. In the above-mentioned study of Borda-Molina et al. [66], however, adding this protease in the amount providing 120,000 PROT units/kg diet increased significantly the AID of all 17 AA measured by an average of 2.6% points. The effect of a commercial product containing viable spores of *B. licheniformis* (ATCC 53757) and its protease (600,000 units/g) on the AA apparent ileal digestibility in Polish WF pea cv. Muza for 23-day-old chickens has lately been examined by Hejdysz et al. [26]. The authors reported increased (*p <* 0.050) digestibility of some AA (Arg, Leu, Phe, Tyr, Ala, Pro) in the presence of this enzyme product (0.5 g/kg diet), with magnitudes in the improvements in AID of Arg, Leu and Pro similar to those seen in the current experiment for the SID of these amino acids determined in 14-day-old birds fed protease-supplemented Tarchalska pea-based diet. However it is worth noting that for the determination of the AA digestibilities these researchers used the ‘substitution’ method in which the assay diet was composed of 30% of pea seeds and 70% of basal feed formulated from maize, SBM and fish meal as the main feedstuffs.

## 5. Conclusions

The results from the current digestibility study indicated an unequal ability of broiler chickens at 14 days of life to digest protein and absorb AA from the tested peas and the presence of polyphenols, particularly condensed tannins, could substantially contribute to such differences. The present results also suggest that when the SID coefficients are taken into account, with the exception of methionine and cysteine, there is no significant difference between these two cultivars of pea in the digestibility of AA in 28-day-old chickens. Generally, considering age-related differences in the SID values of IAA between cultivars, the peas tested were ranked as follows: day 14, CF Milwa was less than WF Tarchalska; day 28, CF Milwa was equal to WF Tarchalska, but within varietal types these differences were negligible. Therefore, when a digestible AA system is used, the SID coefficients of indispensable AA determined at 14 days of age in low-tannin WF peas are not applicable to the formulation of grower-type feed mixtures containing seeds of coloured-flowered cultivars. The commercial mono-component protease selected for applying in this study had a beneficial effect on the SID of AA, and the magnitude of improvement in individual AA digestibility to this product varied depending on pea cultivar and bird age. However, it appears that this type of microbial protease may be successfully used to increase the overall quantity of nutritionally essential AA available for absorption from low-tannin white-flowered peas fed to bids at both ages, and from coloured-flowered peas when fed to the physiologically more mature chickens aged 28 days.

## Figures and Tables

**Table 1:** Composition (g/kg as fed) of nitrogen-free diet used to estimate basal ileal endogenous amino acid losses and the assay diets for the determination of apparent ileal amino acid digestibility of the pea variants.

Ingredient	N-Free Diet	Pea-Based Assay Diets ^1^	
		WF	CF
Test pea	-	810	710
Maize starch	200	50	100
Dextrose	647	50	100
Cellulose	50	-	-
Refined rapeseed oil	40	40	40
Ca(H_2_PO_4_)_2_	19	19	19
Limestone	18	16	16
NaHCO_3_	7	2	2
Vitamin-trace mineral premix ^2^	5	5	5
KCl	3	-	-
K_2_CO_3_	2	-	-
NaCl	2	3	3
MgO	2	-	-
Indigestible marker (Cr_2_O_3_)	5	5	5

^1^ The diets were offered without or with the microbial serine protease product added on top (200 mg/kg). ^2^ Premix (DSM Nutritional Products Ltd., Mszczonów, Poland) provided per kilogram of diet: retinol, 3600 μg; cholecalciferol, 125 μg; α-tocopherol, 45 mg; menadione, 3 mg; thiamine, 3.25 mg; riboflavin, 7.5 mg; pyridoxine, 5 mg; cobalamin, 0.03 mg; biotin 0.25 mg; folic acid, 1.5 mg; nicotinic acid, 45 mg; calcium pantothenate, 15 mg; choline chloride, 1000 mg; Fe, 67.5 mg; Zn, 75 mg; Mn, 100 mg; Cu, 17.5 mg; I, 1 mg; Se, 0.28 mg. WF = white-flowered pea cv. Tarchalska; CF = coloured-flowered pea cv. Milwa.

**Table 2:** Analysed composition ^1^ of test peas.

Component	Pea Variant	
	White-Flowered cv. Tarchalska (WF)	Coloured-Flowered cv. Milwa (CF)
Dry matter (DM), %	88.4	89.9
Crude nutrients, g/kg DM		
Crude protein (N × 6.25)	223.0	251.0
Crude fibre	58.2	65.4
Amino acids, g/kg DM		
Alanine (Ala)	9.3	9.7
Arginine (Arg)	22.3	21.9
Aspartic acid (Asp)	25.1	28.8
Cysteine (Cys)	3.3	3.2
Glutamic acid (Glu)	39.2	46.9
Glycine (Gly)	9.9	9.8
Histidine (His)	7.1	6.4
Isoleucine (Ile)	9.2	8.6
Leucine (Leu)	15.7	16.0
Lysine (Lys)	19.1	17.6
Methionine (Met)	2.7	2.2
Phenylalanine (Phe)	11.7	11.4
Proline (Pro)	8.9	9.6
Serine (Ser)	11.4	10.7
Threonine (Thr)	8.5	8.1
Tyrosine (Tyr)	7.1	8.7
Valine (Val)	10.1	9.9
Total amino acids ^2^	220.6	229.5
Indispensable amino acids ^3^	106.4	102.1
Trypsin inhibitor activity ^4^	1.79	1.77
Tannins		
Total phenolics ^5^	0.81	3.50
Condensed tannins ^6^	0.14	0.63

^1^ Data are based on duplicate analyses. ^2^ Sum of all the amino acids listed above. ^3^ Sum of Arg, His, Ile, Leu, Lys, Met, Phe, Thr and Val—the amino acids, along with tryptophan, traditionally classified as nutritionally essential for poultry. ^4^ mg pure trypsin inhibited per g DM of sample. ^5^ g tannic acid equivalents/kg DM. ^6^ g catechin equivalents/kg DM.

**Table 3:** Basal ileal flow of endogenous amino acids (mg/kg DMI) in broiler chickens fed the N-free diet at 14 and 28 days age (*n* = 6).

Amino Acid	14 d	28 d	*p*-Value ^1^
Ala	276 (33)	194 (25)	<0.001
Arg	271 (41)	181 (25)	<0.001
Asp	385 (36)	296 (20)	<0.001
Cys	118 (6)	126 (9)	0.094
Glu	490 (55)	379 (66)	0.010
Gly	266 (34)	210 (24)	0.009
His	108 (4)	89 (8)	<0.001
Ile	259 (34)	234 (27)	0.193
Leu	330 (20)	285 (37)	0.025
Lys	251 (15)	174 (20)	<0.001
Met	77 (5)	73 (7)	0.286
Phe	263 (37)	199 (19)	0.003
Pro	280 (20)	255 (24)	0.081
Ser	289 (14)	233 (19)	<0.001
Thr	352 (26)	334 (39)	0.362
Tyr	232 (17)	205 (12)	0.009
Val	300 (38)	230 (28)	0.004
Total amino acids ^2^	4545 (279)	3696 (355)	<0.001

^1^*p*-value of *t*-test. ^2^ Sum of all the amino acids listed above. Figures in parentheses are standard deviations.

**Table 4:** Flower colour variant, age of broiler chickens and protease addition main effects on the standardised ileal amino acid digestibility of test pea seeds.

Amino Acid	Main Effect Means (*n* = 24)						Pooled SEM	Source of Variation (*p*-Value)				
	Pea Variant (V)		Age (A)		Protease (P)							
	WF	CF	14 d	28 d	−	+		V	A	P	V × A	V × P ^1^
Ala	0.836	0.831	0.827	0.840	0.822	0.845	0.0048	0.544	0.152	0.012	0.198	0.114
Arg	0.887	0.894	0.883	0.897	0.875	0.906	0.0047	0.346	0.056	0.000	0.001	0.499
Asp	0.855	0.843	0.839	0.859	0.838	0.860	0.0045	0.126	0.009	0.010	0.031	0.045
Cys	0.799	0.708	0.754	0.753	0.738	0.769	0.0082	0.000	0.996	0.002	0.897	0818
Glu	0.892	0.864	0.869	0.887	0.868	0.888	0.0049	0.001	0.024	0.021	0.000	0.092
Gly	0.832	0.822	0.816	0.838	0.813	0.841	0.0050	0.313	0.018	0.002	0.612	0.295
His	0.854	0.844	0.846	0.851	0.841	0.857	0.0039	0.164	0.498	0.036	0.118	0.109
Ile	0.808	0.789	0.792	0.814	0.789	0.817	0.0056	0.299	0.028	0.007	0.048	0.046
Leu	0.834	0.807	0.813	0.828	0.805	0.836	0.0058	0.005	0.130	0.002	0.009	0.131
Lys	0.880	0.863	0.862	0.881	0.856	0.887	0.0052	0.058	0.030	0.001	0.047	0.831
Met	0.865	0.785	0.816	0.833	0.811	0.838	0.0090	0.000	0.215	0.048	0.538	0.307
Phe	0.852	0.840	0.839	0.853	0.835	0.857	0.0047	0.199	0.147	0.014	0.039	0.305
Pro	0.828	0.819	0.808	0.839	0.806	0.841	0.0063	0.415	0.003	0.001	0.030	0.158
Ser	0.813	0.826	0.812	0.828	0.809	0.830	0.0046	0.124	0.064	0.018	0.471	0.080
Thr	0.802	0.824	0.795	0.832	0.795	0.832	0.0063	0.052	0.001	0.001	0.742	0.145
Tyr	0.851	0.857	0.847	0.861	0.845	0.863	0.0041	0.519	0.065	0.023	0.426	0.211
Val	0.826	0.812	0.812	0.825	0.805	0.833	0.0051	0.114	0.142	0.003	0.157	0.106
17AA	0.842	0.826	0.825	0.843	0.821	0.847	0.0044	0.027	0.021	0.001	0.057	0.132
IAA	0.845	0.830	0.829	0.846	0.823	0.852	0.0045	0.036	0.022	0.000	0.035	0.150

^1^ The remaining interactions (A × P, V × A × P) were not statistically significant for any of the amino acids (*p* ≥ 0.090). ‘−’ = without enzyme; ‘+’ = with enzyme; WF = white-flowered pea cv. Tarchalska; CF = coloured-flowered pea cv. Milwa; SEM = standard error of the mean; 17AA = average digestibility of all amino acids analysed; IAA = average digestibility of nine indispensable amino acids (Arg, His, Ile, Leu, Lys, Met, Phe, Thr and Val); d = days.

**Table 5:** Results of contrast testing between the standardised ileal amino acids digestibility of WF and CF peas ^1^ fed without protease supplementation (*n* = 6) in broiler chickens at 14 or 28 days of age.

Amino Acid	Contrasts WF vs. CF			
	*p*-Values for 14 d	Difference ^2^	*p*-Values for 28 d	Difference ^2^
Ala	0.135	2.8	0.484	1.3
Arg	0.813	0.4	0.068	−2.7
Asp	0.008	4.1	0.389	1.3
Cys	0.000	9.4	0.000	8.2
Glu	0.000	7.0	0.452	1.1
Gly	0.140	2.7	0.566	1.0
His	0.048	3.0	0.305	1.5
Ile	0.042	4.1	0.314	2.0
Leu	0.002	6.0	0.205	2.4
Lys	0.037	3.8	0.986	0.1
Met	0.000	10.0	0.000	8.8
Phe	0.016	4.3	0.868	−0.3
Pro	0.053	4.1	0.792	0.6
Ser	0.911	0.2	0.899	0.2
Thr	0.877	−0.3	0.571	−1.2
Tyr	0.704	0.6	0.811	0.3
Val	0.038	3.9	0.264	2.1
17AA	0.009	3.9	0.257	1.2
IAA	0.011	3.8	0.342	1.4

^1^ WF = white-flowered pea cv. Tarchalska, CF = coloured-flowered pea cv. Milwa. ^2^ between the SID value for WF pea and the SID value for CF pea, in percentage points. 17AA = average digestibility of all amino acids analysed; IAA = average digestibility of nine indispensable amino acids (Arg, His, Ile, Leu, Lys, Met, Phe, Thr, Val).

**Table 6:** Standardised ileal digestibility of amino acids in the seeds of the white-flowered pea cv. Tarchalska as affected by chicken age (14 or 28 d) and supplementation of assay diet with protease.

Amino Acid	Means for Treatment Combinations (*n* = 6)				Pooled SEM	*p*-Values for Main Effects Contrasts (*n* = 12)	
	14 d −	14 d +	28 d −	28 d +		Age	Protease
Ala	0.828	0.843	0.836	0.838	0.0040	0.866	0.276
Arg	0.867 ^c^	0.919 ^a^	0.871 ^c^	0.891 ^b^	0.0047	0.006	0.000
Asp	0.848	0.857	0.856	0.857	0.0021	0.357	0.231
Cys	0.790 ^a, b^	0.806 ^a, b^	0.775 ^b^	0.824 ^a^	0.0065	0.922	0.011
Glu	0.898 ^a^	0.897 ^a^	0.881 ^b^	0.892 ^a, b^	0.0025	0.025	0.283
Gly	0.817 ^b^	0.830 ^b^	0.827 ^b^	0.854 ^a^	0.0046	0.041	0.020
His	0.855	0.860	0.850	0.852	0.0035	0.372	0.602
Ile	0.796	0.817	0.813	0.806	0.0037	0.672	0.289
Leu	0.826 ^b^	0.854 ^a^	0.825 ^b^	0.832 ^b^	0.0038	0.095	0.014
Lys	0.866 ^b^	0.893 ^a^	0.866 ^b^	0.896 ^a^	0.0039	0.764	0.000
Met	0.858	0.863	0.858	0.881	0.0079	0.605	0.421
Phe	0.854	0.855	0.836	0.862	0.0048	0.626	0.176
Pro	0.807 ^b^	0.840 ^a^	0.828 ^a, b^	0.836 ^a^	0.0051	0.360	0.037
Ser	0.803	0.814	0.818	0.818	0.0037	0.207	0.462
Thr	0.777 ^c^	0.794 ^b^	0.806 ^b^	0.832 ^a^	0.0059	0.001	0.026
Tyr	0.849	0.847	0.846	0.865	0.0042	0.344	0.341
Val	0.819	0.833	0.821	0.833	0.0039	0.952	0.111
17AA	0.833 ^c^	0.848 ^a, b^	0.836 ^b, c^	0.851 ^a^	0.0028	0.554	0.006
IAA	0.835 ^b^	0.854 ^a^	0.838 ^b^	0.854 ^a^	0.0029	0.778	0.002

‘−’ = without enzyme; ‘+’ = with enzyme; SEM = pooled standard error of the mean; 17AA = average digestibility of all amino acids analysed; IAA = average digestibility of nine indispensable amino acids (Arg, His, Ile, Leu, Lys, Met, Phe, Thr, Val). ^a–c^ Different superscript letters within rows indicate significant contrasts (*p <* 0.050) between treatment combination means.

**Table 7:** Standardised ileal digestibility of amino acids in the seeds of the coloured-flowered pea cv. Milwa as affected by chicken age (14 or 28 d) and supplementation of assay diet with protease.

Amino Acid	Means for Treatment Combinations (*n* = 6)				Pooled SEM	*p*-Values for Main Effects Contrasts (*n* = 12)	
	14 d −	14 d +	28 d −	28 d +		Age	Protease
Ala	0.800 ^b^	0.836 ^a, b^	0.823 ^a, b^	0.864 ^a^	0.0089	0.136	0.027
Arg	0.863 ^b^	0.885 ^b^	0.898 ^ab^	0.930 ^a^	0.0083	0.010	0.072
Asp	0.807 ^b^	0.842 ^a, b^	0.843 ^a, b^	0.880 ^a^	0.0086	0.018	0.022
Cys	0.696	0.722	0.693	0.723	0.0066	0.932	0.061
Glu	0.828 ^b^	0.854 ^b^	0.870 ^a, b^	0.905 ^a^	0.0088	0.004	0.043
Gly	0.790 ^b^	0.829 ^a, b^	0.817 ^a, b^	0.855 ^a^	0.0089	0.112	0.024
His	0.825 ^b^	0.846 ^a, b^	0.835 ^b^	0.870 ^a^	0.0069	0.203	0.040
Ile	0.755 ^b^	0.799 ^a, b^	0.793 ^a, b^	0.844 ^a^	0.0107	0.034	0.016
Leu	0.766 ^b^	0.807 ^a, b^	0.801 ^a, b^	0.853 ^a^	0.0104	0.031	0.017
Lys	0.828 ^b^	0.860 ^a, b^	0.865 ^a, b^	0.898 ^a^	0.0093	0.035	0.067
Met	0.757 ^b^	0.787 ^a, b^	0.770 ^a, b^	0.825 ^a^	0.0110	0.251	0.065
Phe	0.811 ^b^	0.839 ^a, b^	0.839 ^a, b^	0.873 ^a^	0.0081	0.043	0.041
Pro	0.766 ^b^	0.818 ^a, b^	0.821 ^a, b^	0.872 ^a^	0.0116	0.017	0.012
Ser	0.801 ^b^	0.830 ^a, b^	0.816 ^a, b^	0.859 ^a^	0.0083	0.156	0.027
Thr	0.780 ^b^	0.829 ^a, b^	0.818 ^b^	0.872 ^a^	0.0109	0.037	0.010
Tyr	0.843 ^b^	0.850 ^b^	0.843 ^b^	0.890 ^a^	0.0070	0.118	0.040
Val	0.780 ^b^	0.816 ^a, b^	0.800 ^b^	0.850 ^a^	0.0094	0.121	0.016
17AA	0.794 ^b^	0.826 ^a, b^	0.820 ^b^	0.863 ^a^	0.0080	0.029	0.011
IAA	0.796 ^b^	0.830 ^a, b^	0.824 ^b^	0.868 ^a^	0.0083	0.025	0.010

‘−’ = without enzyme; ‘+’ = with enzyme; SEM = pooled standard error of the mean; 17AA = average digestibility of all amino acids analysed; IAA = average digestibility of nine indispensable amino acids (Arg, His, Ile, Leu, Lys, Met, Phe, Thr, Val). ^a, b^ Different superscript letters within rows indicate significant contrasts (*p <* 0.050) between treatment combination means.

## Supplementary Materials

The following are available online at https://www.mdpi.com/2076-2615/10/11/2099/s1, Table S1: Raw data for apparent ileal digestibility (AID) of amino acids in the white-flowered pea cv. Tarchalska, Table S2: Raw data for apparent ileal digestibility (AID) of amino acids in the coloured-flowered pea cv. Milwa.
