# Supplementary material for: Standardised Ileal Amino Acid Digestibility in Field Pea Seeds of Two Cultivars Differing in Flower Colour for Broiler Chickens: Effects of Bird Age and Microbial Protease
Source: Animals (Basel). 2020 Nov 12;10(11):2099. doi: 10.3390/ani10112099 (PMC7697707; doi:10.3390/ani10112099)
Supplement: Supplementary file 1 [file animals-10-02099-s001.pdf]

Supplementary Table S1. Raw data for apparent ileal digestibility (AID) of amino acids in the white-flowered pea cv. Tarchalska.

Amino Acid	Treatment Combinations: Age of Broilers (14 or 28 d) by Protease Addition ('–' without or '+' with enzyme)			
	14 d –	14 d +	28 d –	28 d +
Asp	0.831370	0.832509	0.836655	0.841264
	0.836087	0.850148	0.847080	0.853434
	0.835107	0.825366	0.849988	0.829341
	0.837367	0.825038	0.842328	0.848030
	0.828359	0.854281	0.847921	0.836036
	0.814395	0.845669	0.829989	0.849107
Thr	0.744985	0.709605	0.741036	0.776466
	0.753203	0.756586	0.728665	0.824281
	0.719728	0.732086	0.769844	0.766148
	0.729716	0.727925	0.772362	0.770893
	0.707889	0.774555	0.786814	0.783051
	0.727037	0.771618	0.774228	0.797154
Ser	0.776159	0.746393	0.789280	0.804225
	0.785603	0.789622	0.765917	0.787855
	0.759725	0.778805	0.799021	0.780443
	0.807110	0.770112	0.796082	0.794798
	0.745660	0.811404	0.804796	0.806443
	0.756998	0.801546	0.805303	0.785222
Glu	0.877789	0.858994	0.884196	0.875530
	0.881328	0.893958	0.853322	0.885067
	0.868632	0.875837	0.877383	0.870111
	0.900951	0.875155	0.869107	0.884474
	0.883680	0.896655	0.873189	0.885601
	0.887464	0.888683	0.859190	0.881297
Pro	0.773598	0.765898	0.811601	0.783885
	0.772007	0.804663	0.788978	0.801061
	0.754100	0.794693	0.814889	0.763236
	0.815588	0.795096	0.791431	0.829096
	0.747626	0.829960	0.794065	0.813538
	0.761153	0.826683	0.768406	0.824252
Gly	0.792795	0.762756	0.796421	0.811024
	0.793802	0.809693	0.781199	0.848702
	0.774180	0.787765	0.813240	0.845356
	0.823182	0.784363	0.807130	0.809868
	0.755812	0.821199	0.812821	0.824700
	0.767504	0.817339	0.798374	0.830185
Ala	0.801542	0.770245	0.808476	0.822853
	0.803457	0.819531	0.784842	0.802415
	0.782656	0.796940	0.824367	0.807265
	0.831280	0.797372	0.811938	0.817832
	0.761286	0.832822	0.827636	0.833231
	0.775554	0.830054	0.807990	0.796374
Val	0.791768	0.763921	0.798567	0.798476
	0.794020	0.810964	0.770562	0.798728
	0.776318	0.788047	0.805342	0.783579
	0.821236	0.783703	0.797654	0.806434
	0.754533	0.821963	0.805123	0.815841
	0.768816	0.816932	0.785862	0.831190
Ile	0.758572	0.741102	0.782699	0.783181
	0.754425	0.792950	0.754958	0.777435
	0.762854	0.772511	0.797925	0.770500
	0.766547	0.767813	0.781225	0.794609

	0.749327	0.808768	0.794313	0.750352
	0.770980	0.803977	0.773849	0.766277
Leu	0.803907	0.811890	0.810095	0.803179
	0.811610	0.824517	0.778598	0.830813
	0.792310	0.838705	0.820314	0.789100
	0.804436	0.798214	0.800929	0.812456
	0.790749	0.833998	0.817078	0.803587
	0.803175	0.860120	0.795098	0.819437
Tyr	0.805904	0.778161	0.812157	0.821271
	0.800629	0.826131	0.808942	0.845498
	0.800253	0.788896	0.843663	0.845873
	0.826394	0.794960	0.814708	0.862658
	0.807918	0.839159	0.819590	0.813974
	0.833934	0.831207	0.786195	0.807026
Phe	0.826690	0.789461	0.832719	0.809244
	0.832523	0.834339	0.799875	0.837830
	0.816083	0.818778	0.834062	0.834343
	0.846062	0.815983	0.814238	0.858027
	0.834428	0.848532	0.819882	0.889953
	0.795213	0.842500	0.784144	0.806432
His	0.839487	0.800536	0.839375	0.826537
	0.843983	0.844560	0.817060	0.841343
	0.827403	0.828961	0.851810	0.836718
	0.855719	0.829822	0.825598	0.857511
	0.806213	0.860944	0.836389	0.813108
	0.816544	0.856030	0.814556	0.819139
Lys	0.836854	0.841785	0.843586	0.868270
	0.839679	0.884887	0.850662	0.895552
	0.859054	0.870380	0.850550	0.867191
	0.861074	0.869349	0.866659	0.876749
	0.854276	0.891390	0.846816	0.905217
	0.835367	0.884354	0.859866	0.886426
Arg	0.848348	0.883543	0.864049	0.881019
	0.846647	0.913642	0.862378	0.893490
	0.840646	0.897324	0.866621	0.864348
	0.854973	0.896759	0.859135	0.881648
	0.848862	0.916588	0.860850	0.880680
	0.861921	0.909396	0.847767	0.881376
Cys	0.715249	0.747537	0.773506	0.789170
	0.729605	0.806551	0.695307	0.778702
	0.764463	0.718331	0.711639	0.806696
	0.769103	0.760878	0.737223	0.718266
	0.754303	0.764216	0.729623	0.775434
	0.732566	0.733903	0.707284	0.745533
Met	0.785576	0.829278	0.864981	0.883675
	0.782837	0.867572	0.792733	0.871930
	0.844603	0.753343	0.843805	0.893143
	0.849846	0.877508	0.816204	0.773836
	0.878662	0.818830	0.837432	0.865336
	0.822363	0.816382	0.820397	0.789013

Supplementary Table S2. Raw data for apparent ileal digestibility (AID) of amino acids in the coloured-flowered pea cv. Milwa.

Amino Acid	Treatment Combinations: Age of Broilers (14 or 28 d) by Protease Addition ('–' without or '+' with enzyme)			
	14 d –	14 d +	28 d –	28 d +
Asp	0.691321	0.874390	0.852558	0.887679
	0.808066	0.819144	0.829904	0.865535
	0.759682	0.784275	0.834932	0.872783
	0.855606	0.809411	0.812276	0.836460
	0.829153	0.830032	0.810269	0.892579
	0.786632	0.823045	0.832707	0.841766
Thr	0.630093	0.847432	0.795991	0.861312
	0.760117	0.752218	0.767351	0.831220
	0.681811	0.724205	0.786775	0.847137
	0.808918	0.753549	0.755603	0.770393
	0.770510	0.803365	0.751855	0.852906
	0.735361	0.797393	0.770806	0.790714
Ser	0.675825	0.839875	0.817266	0.875524
	0.778043	0.790561	0.788574	0.828455
	0.743544	0.762891	0.790450	0.855773
	0.822717	0.781681	0.771207	0.771930
	0.806133	0.798903	0.765275	0.852479
	0.758732	0.783638	0.785649	0.790258
Glu	0.716197	0.888522	0.884367	0.918489
	0.829232	0.843313	0.858461	0.887725
	0.789736	0.801442	0.866066	0.904610
	0.869672	0.811291	0.836816	0.862918
	0.855407	0.842371	0.838281	0.914435
	0.804664	0.835226	0.852071	0.863728
Pro	0.611612	0.849062	0.811397	0.867293
	0.759162	0.757583	0.772024	0.838742
	0.686326	0.727392	0.802673	0.856670
	0.812332	0.757626	0.780081	0.790168
	0.765057	0.798608	0.775754	0.864670
	0.733930	0.791103	0.786393	0.803865
Gly	0.655661	0.849928	0.821694	0.859746
	0.778285	0.781771	0.789031	0.832436
	0.718420	0.756709	0.804374	0.841458
	0.823459	0.772033	0.778111	0.785893
	0.794570	0.808658	0.775092	0.855611
	0.762229	0.802270	0.767982	0.794867
Ala	0.664676	0.852379	0.839133	0.871315
	0.780520	0.790205	0.792371	0.842058
	0.730825	0.764807	0.812199	0.851343
	0.829322	0.772296	0.778373	0.792201
	0.807586	0.812595	0.778342	0.866455
	0.765359	0.802065	0.779518	0.801250
Val	0.641926	0.837159	0.813494	0.851544
	0.762794	0.771599	0.764634	0.822465
	0.707315	0.739486	0.781776	0.836980
	0.811721	0.753772	0.752055	0.776369
	0.787918	0.792219	0.751213	0.854045
	0.744010	0.779789	0.762853	0.789872
Ile	0.606007	0.824916	0.805213	0.842702
	0.739775	0.755616	0.753466	0.813412
	0.677454	0.721196	0.769789	0.825872
	0.796606	0.733640	0.740400	0.760125

	0.768396	0.776425	0.738760	0.842364
	0.718950	0.762371	0.747785	0.778371
Leu	0.624706	0.839649	0.820025	0.860719
	0.753774	0.777189	0.773792	0.828824
	0.705876	0.740554	0.789761	0.842517
	0.813284	0.750263	0.755489	0.784535
	0.793872	0.788441	0.755239	0.862025
	0.738631	0.779320	0.768007	0.794015
Tyr	0.730447	0.859765	0.842653	0.869423
	0.823373	0.804702	0.794742	0.855598
	0.786026	0.781583	0.795311	0.866223
	0.843750	0.773391	0.794222	0.823468
	0.834443	0.820364	0.796950	0.874816
	0.812065	0.810483	0.830259	0.832206
Phe	0.687022	0.854891	0.862990	0.872704
	0.803459	0.810329	0.811781	0.850489
	0.755701	0.778223	0.821145	0.857273
	0.840859	0.788485	0.796434	0.818385
	0.820709	0.815819	0.794791	0.877421
	0.777568	0.805510	0.809531	0.826190
His	0.737743	0.859820	0.888860	0.873763
	0.812774	0.817831	0.803494	0.851965
	0.781481	0.801458	0.821893	0.860266
	0.850258	0.809756	0.788177	0.814763
	0.823870	0.831317	0.788874	0.870034
	0.803134	0.814898	0.800028	0.832481
Lys	0.700589	0.897004	0.899735	0.913751
	0.830629	0.849849	0.850112	0.891381
	0.791621	0.804334	0.866491	0.901248
	0.875689	0.810426	0.829988	0.853993
	0.857043	0.848063	0.830216	0.912213
	0.801966	0.839000	0.838260	0.840450
Arg	0.745421	0.917758	0.914206	0.935067
	0.860566	0.883331	0.888205	0.910814
	0.832059	0.829532	0.888856	0.924750
	0.899823	0.835265	0.870358	0.899399
	0.895186	0.879137	0.875494	0.938086
	0.842071	0.865034	0.884068	0.901815
Cys	0.636091	0.571787	0.647932	0.688930
	0.649560	0.706885	0.643202	0.688889
	0.624596	0.648297	0.638822	0.653309
	0.666550	0.759150	0.639262	0.696228
	0.657180	0.673226	0.637833	0.655987
	0.658385	0.670891	0.642955	0.632603
Met	0.695344	0.662563	0.671778	0.797128
	0.717345	0.770522	0.732002	0.788087
	0.760415	0.765425	0.800729	0.803952
	0.772377	0.730462	0.778072	0.788377
	0.593203	0.789627	0.646104	0.776693
	0.779493	0.784516	0.781153	0.788678
